# Dibromido(2,4,6-tri-2-pyridyl-1,3,5-triazine-κ^3^ *N* ^2^,*N* ^1^,*N* ^6^)manganese(II)

**DOI:** 10.1107/S1600536811045211

**Published:** 2011-11-05

**Authors:** Kwang Ha

**Affiliations:** a School of Applied Chemical Engineering, The Research Institute of Catalysis, Chonnam National University, Gwangju 500-757, Republic of Korea

## Abstract

The Mn^II^ ion in the title complex, [MnBr_2_(C_18_H_12_N_6_)], is five-coordinated in a distorted square-pyramidal geometry by three N atoms of the tridentate 2,4,6-tri-2-pyridyl-1,3,5-triazine (tptz) ligand and two bromide anions. In the crystal, the pyridyl rings coordinated to the Mn atom are inclined slightly to their carrier triazine ring [dihedral angles = 8.0 (3) and 7.5 (3)°], whereas the uncoordinated pyridyl ring is located approximately parallel to the triazine ring [dihedral angle = 3.7 (3)°]. The complexes are stacked in columns along the *a* axis and linked by inter­molecular C—H⋯Br hydrogen bonds, forming chains. In the column, inter­molecular π–π inter­actions between the six-membered rings are present, the shortest centroid–centroid distance being 3.750 (4) Å.

## Related literature

For the crystal structure of the related compound [MnBr_2_(tptz)(H_2_O)]·H_2_O, see: Ha (2011 ▶).
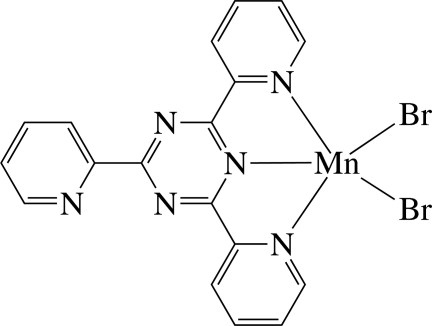


## Experimental

### 

#### Crystal data

[MnBr_2_(C_18_H_12_N_6_)]
*M* *_r_* = 527.10
Triclinic, 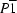

*a* = 8.7095 (19) Å
*b* = 10.498 (2) Å
*c* = 11.213 (3) Å
α = 110.094 (4)°
β = 98.471 (4)°
γ = 91.820 (5)°
*V* = 948.5 (4) Å^3^
*Z* = 2
Mo *K*α radiation
μ = 4.92 mm^−1^
*T* = 200 K
0.27 × 0.17 × 0.09 mm

#### Data collection

Bruker SMART 1000 CCD diffractometer
Absorption correction: multi-scan (*SADABS*; Bruker, 2000 ▶) *T*_min_ = 0.695, *T*_max_ = 1.000
6897 measured reflections
4548 independent reflections
3124 reflections with *I* > 2σ(*I*)
*R*_int_ = 0.024

#### Refinement

*R*[*F* ^2^ > 2σ(*F* ^2^)] = 0.046
*wR*(*F* ^2^) = 0.114
*S* = 1.14
4548 reflections
244 parameters
H-atom parameters constrained
Δρ_max_ = 0.90 e Å^−3^
Δρ_min_ = −1.00 e Å^−3^

### 

Data collection: *SMART* (Bruker, 2000 ▶); cell refinement: *SAINT* (Bruker, 2000 ▶); data reduction: *SAINT*; program(s) used to solve structure: *SHELXS97* (Sheldrick, 2008 ▶); program(s) used to refine structure: *SHELXL97* (Sheldrick, 2008 ▶); molecular graphics: *ORTEP-3* (Farrugia, 1997 ▶) and *PLATON* (Spek, 2009 ▶); software used to prepare material for publication: *SHELXL97*.

## Supplementary Material

Crystal structure: contains datablock(s) global, I. DOI: 10.1107/S1600536811045211/zq2132sup1.cif

Structure factors: contains datablock(s) I. DOI: 10.1107/S1600536811045211/zq2132Isup2.hkl

Additional supplementary materials:  crystallographic information; 3D view; checkCIF report

## Figures and Tables

**Table:** 

Mn1—N1	2.181 (4)
Mn1—N4	2.314 (5)
Mn1—N6	2.331 (4)
Mn1—Br2	2.4884 (11)
Mn1—Br1	2.4957 (11)

**Table:** 

N1—Mn1—N4	70.43 (15)
N1—Mn1—N6	71.07 (16)
Br2—Mn1—Br1	111.10 (4)

**Table 2:** Hydrogen-bond geometry (Å, °)

*D*—H⋯*A*	*D*—H	H⋯*A*	*D*⋯*A*	*D*—H⋯*A*
C10—H10⋯Br1^i^	0.95	2.91	3.782 (6)	153
C15—H15⋯Br1^ii^	0.95	2.91	3.744 (6)	148

Symmetry codes: (i) 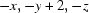; (ii) 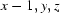.

**Fig. 1.**
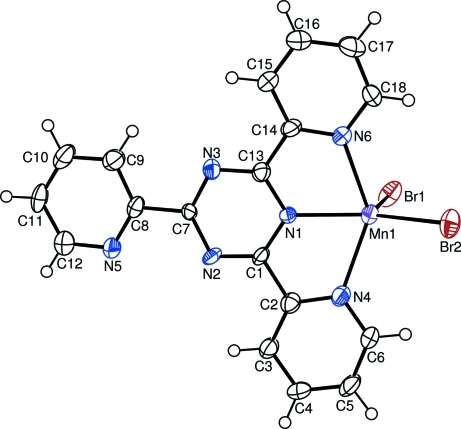
The structure of the title complex, with displacement ellipsoids drawn at the 50% probability level; H atoms are shown as small circles of arbitrary radius.

**Fig. 2.**
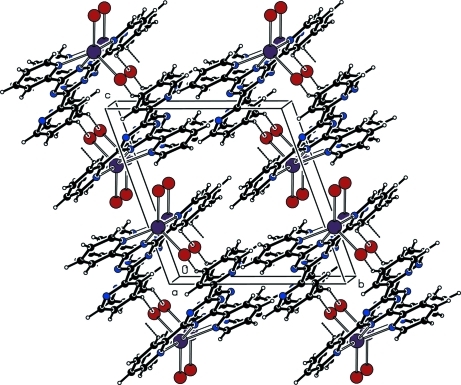
View of the unit-cell contents of the title complex. Hydrogen-bond interactions are drawn with dashed lines.

## supplementary crystallographic information

### Comment

In the title complex, [MnBr_2_(tptz)] (tptz = 2,4,6-tri-2-pyridyl-1,3,5-triazine, C_18_H_12_N_6_), the Mn^II^ ion is five-coordinated in a distorted square-pyramidal geometry by three N atoms of the tridentate tptz ligand and two bromide anions (Fig. 1). By contrast, in the previously reported analogous complex [MnBr_2_(tptz)(H_2_O)].H_2_O, obtained from a CH_3_CN solution, the Mn^II^ ion is six-coordinated in a distorted octahedral environment by three N atoms, two Br atoms and one O atom from the water ligand (Ha, 2011).

While the Mn—Br bond lengths are almost equal, the Mn—N bond lengths are somewhat different (Table 1). The Mn1—N4/6(pyridyl) bonds are slightly longer than the Mn1—N1(triazine) bond. In the crystal, the pyridyl rings coordinated to the Mn atom are inclined slightly to their carrier triazine ring [dihedral angles = 8.0 (3)° and 7.5 (3)°], whereas the uncoordinated pyridyl ring is located approximately parallel to the triazine ring [dihedral angle = 3.7 (3)°]. The complexes are stacked in columns along the *a* axis and linked by intermolecular C—H···Br hydrogen bonds, forming one-dimensional chains (Fig. 2 and Table 2). In the column, intermolecular π-π interactions between the six-membered rings are present, the shortest centroid-centroid distance being 3.750 (4) Å.

### Experimental

To a solution of MnBr_2_.4H_2_O (0.2868 g, 1.00 mmol) in MeOH (30 ml) was added 2,4,6-tri-2-pyridyl-1,3,5-triazine (0.1561 g, 0.50 mmol) and stirred for 3 h at room temperature. The formed precipitate was separated by filtration and washed with MeOH and dried under vacuum, to give an orange powder (0.1065 g). Crystals suitable for X-ray analysis were obtained by slow evaporation from a dimethyl sulfoxide (DMSO) solution at 90 °C.

### Refinement

H atoms were positioned geometrically and allowed to ride on their respective parent atoms [C—H = 0.95 Å and *U*_iso_(H) = 1.2*U*_eq_(C)]. The highest peak (0.90 e Å^-3^) and the deepest hole (-0.99 e Å^-3^) in the difference Fourier map are located 1.65 Å and 0.80 Å from the atoms H16 and Br1, respectively.

### Crystal data

**Table:**

[MnBr_2_(C_18_H_12_N_6_)]	*Z* = 2
*M**_r_* = 527.10	*F*(000) = 514
Triclinic, *P*1	*D*_x_ = 1.846 Mg m^−^^3^
Hall symbol: -P 1	Mo *K*α radiation, λ = 0.71073 Å
*a* = 8.7095 (19) Å	Cell parameters from 2967 reflections
*b* = 10.498 (2) Å	θ = 2.3–28.3°
*c* = 11.213 (3) Å	µ = 4.92 mm^−^^1^
α = 110.094 (4)°	*T* = 200 K
β = 98.471 (4)°	Block, orange
γ = 91.820 (5)°	0.27 × 0.17 × 0.09 mm
*V* = 948.5 (4) Å^3^	

### Data collection

**Table:**

Bruker SMART 1000 CCD diffractometer	4548 independent reflections
Radiation source: fine-focus sealed tube	3124 reflections with *I* > 2σ(*I*)
graphite	*R*_int_ = 0.024
φ and ω scans	θ_max_ = 28.3°, θ_min_ = 2.0°
Absorption correction: multi-scan (*SADABS*; Bruker, 2000)	*h* = −11→6
*T*_min_ = 0.695, *T*_max_ = 1.000	*k* = −12→14
6897 measured reflections	*l* = −14→14

### Refinement

**Table:**

Refinement on *F*^2^	Primary atom site location: structure-invariant direct methods
Least-squares matrix: full	Secondary atom site location: difference Fourier map
*R*[*F*^2^ > 2σ(*F*^2^)] = 0.046	Hydrogen site location: inferred from neighbouring sites
*wR*(*F*^2^) = 0.114	H-atom parameters constrained
*S* = 1.14	*w* = 1/[σ^2^(*F*_o_^2^) + (0.0037*P*)^2^ + 4.4503*P*] where *P* = (*F*_o_^2^ + 2*F*_c_^2^)/3
4548 reflections	(Δ/σ)_max_ = 0.001
244 parameters	Δρ_max_ = 0.90 e Å^−^^3^
0 restraints	Δρ_min_ = −1.00 e Å^−^^3^

### Special details

**Table:**

Geometry. All e.s.d.'s (except the e.s.d. in the dihedral angle between two l.s. planes) are estimated using the full covariance matrix. The cell e.s.d.'s are taken into account individually in the estimation of e.s.d.'s in distances, angles and torsion angles; correlations between e.s.d.'s in cell parameters are only used when they are defined by crystal symmetry. An approximate (isotropic) treatment of cell e.s.d.'s is used for estimating e.s.d.'s involving l.s. planes.
Refinement. Refinement of *F*^2^ against ALL reflections. The weighted *R*-factor *wR* and goodness of fit *S* are based on *F*^2^, conventional *R*-factors *R* are based on *F*, with *F* set to zero for negative *F*^2^. The threshold expression of *F*^2^ > σ(*F*^2^) is used only for calculating *R*-factors(gt) *etc*. and is not relevant to the choice of reflections for refinement. *R*-factors based on *F*^2^ are statistically about twice as large as those based on *F*, and *R*- factors based on ALL data will be even larger.

### Fractional atomic coordinates and isotropic or equivalent isotropic displacement parameters (Å^2^)

**Table:**

	*x*	*y*	*z*	*U*_iso_*/*U*_eq_	
Mn1	0.60194 (9)	1.09912 (9)	0.35103 (8)	0.0302 (2)	
Br1	0.69262 (7)	1.19838 (8)	0.19802 (6)	0.04687 (19)	
Br2	0.77379 (7)	1.18883 (7)	0.56522 (6)	0.04513 (19)	
N1	0.3893 (5)	0.9697 (4)	0.2464 (4)	0.0269 (9)	
N2	0.2650 (5)	0.7593 (4)	0.0981 (4)	0.0285 (10)	
N3	0.1203 (5)	0.9522 (5)	0.1701 (4)	0.0302 (10)	
N4	0.6578 (5)	0.8741 (5)	0.2753 (4)	0.0302 (10)	
N5	−0.0035 (5)	0.6182 (5)	−0.0683 (5)	0.0377 (11)	
N6	0.3875 (5)	1.2202 (5)	0.4059 (4)	0.0314 (10)	
C1	0.3892 (5)	0.8368 (6)	0.1774 (5)	0.0254 (11)	
C2	0.5412 (6)	0.7819 (6)	0.1970 (5)	0.0296 (12)	
C3	0.5601 (6)	0.6439 (6)	0.1409 (5)	0.0352 (13)	
H3	0.4751	0.5824	0.0868	0.042*	
C4	0.7049 (7)	0.5973 (7)	0.1651 (6)	0.0430 (15)	
H4	0.7213	0.5039	0.1274	0.052*	
C5	0.8253 (7)	0.6917 (7)	0.2461 (6)	0.0435 (15)	
H5	0.9256	0.6635	0.2654	0.052*	
C6	0.7969 (6)	0.8277 (6)	0.2984 (6)	0.0369 (14)	
H6	0.8801	0.8910	0.3533	0.044*	
C7	0.1345 (6)	0.8229 (5)	0.0923 (5)	0.0263 (11)	
C8	−0.0098 (6)	0.7492 (6)	0.0026 (5)	0.0291 (11)	
C9	−0.1432 (6)	0.8194 (6)	−0.0015 (6)	0.0358 (13)	
H9	−0.1421	0.9126	0.0505	0.043*	
C10	−0.2769 (6)	0.7486 (7)	−0.0842 (6)	0.0419 (15)	
H10	−0.3699	0.7926	−0.0902	0.050*	
C11	−0.2733 (7)	0.6134 (7)	−0.1577 (6)	0.0412 (15)	
H11	−0.3630	0.5629	−0.2156	0.049*	
C12	−0.1355 (7)	0.5530 (7)	−0.1450 (6)	0.0465 (16)	
H12	−0.1350	0.4591	−0.1940	0.056*	
C13	0.2490 (6)	1.0202 (5)	0.2444 (5)	0.0275 (11)	
C14	0.2470 (6)	1.1606 (6)	0.3374 (5)	0.0313 (12)	
C15	0.1094 (7)	1.2227 (6)	0.3520 (6)	0.0456 (16)	
H15	0.0128	1.1771	0.3020	0.055*	
C16	0.1168 (8)	1.3530 (7)	0.4413 (8)	0.060 (2)	
H16	0.0247	1.3986	0.4536	0.071*	
C17	0.2589 (8)	1.4160 (7)	0.5124 (7)	0.0547 (19)	
H17	0.2661	1.5054	0.5744	0.066*	
C18	0.3904 (7)	1.3475 (6)	0.4924 (6)	0.0387 (14)	
H18	0.4878	1.3919	0.5417	0.046*	

### Atomic displacement parameters (Å^2^)

**Table:**

	*U*^11^	*U*^22^	*U*^33^	*U*^12^	*U*^13^	*U*^23^
Mn1	0.0218 (4)	0.0322 (5)	0.0322 (4)	−0.0010 (3)	−0.0009 (3)	0.0085 (4)
Br1	0.0296 (3)	0.0690 (5)	0.0512 (4)	0.0037 (3)	0.0040 (3)	0.0339 (4)
Br2	0.0370 (3)	0.0562 (4)	0.0340 (3)	−0.0057 (3)	−0.0062 (2)	0.0114 (3)
N1	0.020 (2)	0.024 (2)	0.030 (2)	−0.0003 (17)	0.0017 (17)	0.0040 (18)
N2	0.025 (2)	0.030 (3)	0.029 (2)	0.0073 (18)	0.0027 (18)	0.0082 (19)
N3	0.020 (2)	0.027 (3)	0.042 (3)	0.0041 (18)	−0.0024 (19)	0.013 (2)
N4	0.0149 (19)	0.037 (3)	0.032 (2)	0.0013 (18)	−0.0042 (17)	0.008 (2)
N5	0.027 (2)	0.036 (3)	0.043 (3)	0.001 (2)	−0.005 (2)	0.009 (2)
N6	0.019 (2)	0.031 (3)	0.038 (3)	0.0010 (18)	0.0027 (18)	0.006 (2)
C1	0.008 (2)	0.041 (3)	0.024 (2)	0.0005 (19)	0.0005 (17)	0.008 (2)
C2	0.026 (3)	0.029 (3)	0.028 (3)	0.000 (2)	0.004 (2)	0.003 (2)
C3	0.030 (3)	0.033 (3)	0.036 (3)	0.002 (2)	0.002 (2)	0.006 (2)
C4	0.038 (3)	0.039 (4)	0.048 (4)	0.017 (3)	0.005 (3)	0.010 (3)
C5	0.028 (3)	0.044 (4)	0.052 (4)	0.011 (3)	−0.003 (3)	0.012 (3)
C6	0.020 (3)	0.045 (4)	0.040 (3)	0.004 (2)	0.000 (2)	0.009 (3)
C7	0.022 (2)	0.026 (3)	0.029 (3)	0.001 (2)	0.000 (2)	0.009 (2)
C8	0.027 (3)	0.030 (3)	0.030 (3)	−0.001 (2)	0.001 (2)	0.012 (2)
C9	0.028 (3)	0.037 (3)	0.043 (3)	0.006 (2)	0.000 (2)	0.017 (3)
C10	0.023 (3)	0.058 (4)	0.046 (4)	0.002 (3)	−0.004 (2)	0.023 (3)
C11	0.027 (3)	0.051 (4)	0.039 (3)	−0.010 (3)	−0.008 (2)	0.012 (3)
C12	0.040 (4)	0.034 (4)	0.053 (4)	−0.006 (3)	0.002 (3)	0.003 (3)
C13	0.018 (2)	0.028 (3)	0.036 (3)	0.003 (2)	0.000 (2)	0.012 (2)
C14	0.029 (3)	0.029 (3)	0.036 (3)	0.004 (2)	0.007 (2)	0.010 (2)
C15	0.022 (3)	0.040 (4)	0.063 (4)	0.005 (2)	0.002 (3)	0.006 (3)
C16	0.044 (4)	0.030 (4)	0.087 (6)	0.011 (3)	0.013 (4)	−0.004 (4)
C17	0.049 (4)	0.036 (4)	0.065 (5)	0.001 (3)	0.013 (3)	−0.001 (3)
C18	0.034 (3)	0.030 (3)	0.045 (3)	−0.004 (2)	0.005 (3)	0.005 (3)

### Geometric parameters (Å, °)

**Table:**

Mn1—N1	2.181 (4)	C4—H4	0.9500
Mn1—N4	2.314 (5)	C5—C6	1.390 (8)
Mn1—N6	2.331 (4)	C5—H5	0.9500
Mn1—Br2	2.4884 (11)	C6—H6	0.9500
Mn1—Br1	2.4957 (11)	C7—C8	1.490 (7)
N1—C1	1.343 (7)	C8—C9	1.399 (8)
N1—C13	1.348 (6)	C9—C10	1.387 (8)
N2—C1	1.337 (6)	C9—H9	0.9500
N2—C7	1.341 (6)	C10—C11	1.378 (9)
N3—C13	1.314 (6)	C10—H10	0.9500
N3—C7	1.359 (7)	C11—C12	1.386 (9)
N4—C6	1.343 (7)	C11—H11	0.9500
N4—C2	1.353 (6)	C12—H12	0.9500
N5—C12	1.335 (7)	C13—C14	1.486 (7)
N5—C8	1.338 (7)	C14—C15	1.387 (8)
N6—C14	1.350 (7)	C15—C16	1.383 (9)
N6—C18	1.352 (7)	C15—H15	0.9500
C1—C2	1.477 (7)	C16—C17	1.379 (9)
C2—C3	1.393 (8)	C16—H16	0.9500
C3—C4	1.390 (8)	C17—C18	1.377 (9)
C3—H3	0.9500	C17—H17	0.9500
C4—C5	1.393 (8)	C18—H18	0.9500
			
N1—Mn1—N4	70.43 (15)	N4—C6—H6	118.3
N1—Mn1—N6	71.07 (16)	C5—C6—H6	118.3
N4—Mn1—N6	137.87 (15)	N2—C7—N3	123.9 (4)
N1—Mn1—Br2	143.00 (12)	N2—C7—C8	120.3 (5)
N4—Mn1—Br2	102.02 (11)	N3—C7—C8	115.6 (4)
N6—Mn1—Br2	98.41 (11)	N5—C8—C9	124.0 (5)
N1—Mn1—Br1	105.80 (12)	N5—C8—C7	117.3 (5)
N4—Mn1—Br1	104.49 (12)	C9—C8—C7	118.6 (5)
N6—Mn1—Br1	101.81 (12)	C10—C9—C8	117.8 (6)
Br2—Mn1—Br1	111.10 (4)	C10—C9—H9	121.1
C1—N1—C13	115.7 (4)	C8—C9—H9	121.1
C1—N1—Mn1	122.8 (3)	C11—C10—C9	119.1 (6)
C13—N1—Mn1	121.5 (3)	C11—C10—H10	120.4
C1—N2—C7	115.2 (4)	C9—C10—H10	120.4
C13—N3—C7	115.9 (4)	C10—C11—C12	118.5 (5)
C6—N4—C2	117.2 (5)	C10—C11—H11	120.8
C6—N4—Mn1	125.8 (4)	C12—C11—H11	120.8
C2—N4—Mn1	116.9 (3)	N5—C12—C11	124.2 (6)
C12—N5—C8	116.3 (5)	N5—C12—H12	117.9
C14—N6—C18	116.7 (5)	C11—C12—H12	117.9
C14—N6—Mn1	116.3 (4)	N3—C13—N1	124.1 (5)
C18—N6—Mn1	126.7 (4)	N3—C13—C14	120.9 (5)
N2—C1—N1	124.3 (4)	N1—C13—C14	114.9 (4)
N2—C1—C2	122.1 (5)	N6—C14—C15	123.6 (5)
N1—C1—C2	113.6 (4)	N6—C14—C13	114.9 (5)
N4—C2—C3	123.0 (5)	C15—C14—C13	121.5 (5)
N4—C2—C1	115.3 (5)	C16—C15—C14	118.1 (6)
C3—C2—C1	121.7 (5)	C16—C15—H15	120.9
C4—C3—C2	119.2 (5)	C14—C15—H15	120.9
C4—C3—H3	120.4	C17—C16—C15	119.3 (6)
C2—C3—H3	120.4	C17—C16—H16	120.3
C3—C4—C5	118.0 (6)	C15—C16—H16	120.3
C3—C4—H4	121.0	C18—C17—C16	119.1 (6)
C5—C4—H4	121.0	C18—C17—H17	120.5
C6—C5—C4	119.2 (5)	C16—C17—H17	120.5
C6—C5—H5	120.4	N6—C18—C17	123.2 (5)
C4—C5—H5	120.4	N6—C18—H18	118.4
N4—C6—C5	123.3 (5)	C17—C18—H18	118.4
			
N4—Mn1—N1—C1	−9.0 (4)	C3—C4—C5—C6	−0.5 (10)
N6—Mn1—N1—C1	−171.5 (4)	C2—N4—C6—C5	0.1 (9)
Br2—Mn1—N1—C1	−93.0 (4)	Mn1—N4—C6—C5	−177.4 (5)
Br1—Mn1—N1—C1	91.1 (4)	C4—C5—C6—N4	0.2 (10)
N4—Mn1—N1—C13	172.9 (4)	C1—N2—C7—N3	−7.0 (8)
N6—Mn1—N1—C13	10.3 (4)	C1—N2—C7—C8	177.1 (5)
Br2—Mn1—N1—C13	88.9 (4)	C13—N3—C7—N2	7.4 (8)
Br1—Mn1—N1—C13	−87.0 (4)	C13—N3—C7—C8	−176.5 (5)
N1—Mn1—N4—C6	−176.0 (5)	C12—N5—C8—C9	−1.3 (9)
N6—Mn1—N4—C6	−151.0 (4)	C12—N5—C8—C7	178.0 (5)
Br2—Mn1—N4—C6	−33.8 (5)	N2—C7—C8—N5	1.4 (8)
Br1—Mn1—N4—C6	82.0 (5)	N3—C7—C8—N5	−174.9 (5)
N1—Mn1—N4—C2	6.5 (4)	N2—C7—C8—C9	−179.4 (5)
N6—Mn1—N4—C2	31.5 (5)	N3—C7—C8—C9	4.3 (7)
Br2—Mn1—N4—C2	148.8 (4)	N5—C8—C9—C10	0.1 (9)
Br1—Mn1—N4—C2	−95.4 (4)	C7—C8—C9—C10	−179.1 (5)
N1—Mn1—N6—C14	−8.0 (4)	C8—C9—C10—C11	0.2 (9)
N4—Mn1—N6—C14	−32.9 (5)	C9—C10—C11—C12	0.6 (9)
Br2—Mn1—N6—C14	−151.4 (4)	C8—N5—C12—C11	2.2 (10)
Br1—Mn1—N6—C14	94.9 (4)	C10—C11—C12—N5	−1.9 (10)
N1—Mn1—N6—C18	177.2 (5)	C7—N3—C13—N1	0.3 (8)
N4—Mn1—N6—C18	152.3 (4)	C7—N3—C13—C14	−177.6 (5)
Br2—Mn1—N6—C18	33.8 (5)	C1—N1—C13—N3	−7.6 (8)
Br1—Mn1—N6—C18	−79.9 (5)	Mn1—N1—C13—N3	170.7 (4)
C7—N2—C1—N1	−1.2 (7)	C1—N1—C13—C14	170.5 (5)
C7—N2—C1—C2	178.8 (5)	Mn1—N1—C13—C14	−11.2 (6)
C13—N1—C1—N2	8.0 (7)	C18—N6—C14—C15	−0.2 (9)
Mn1—N1—C1—N2	−170.2 (4)	Mn1—N6—C14—C15	−175.6 (5)
C13—N1—C1—C2	−171.9 (5)	C18—N6—C14—C13	−179.4 (5)
Mn1—N1—C1—C2	9.8 (6)	Mn1—N6—C14—C13	5.3 (6)
C6—N4—C2—C3	0.0 (8)	N3—C13—C14—N6	−178.6 (5)
Mn1—N4—C2—C3	177.7 (4)	N1—C13—C14—N6	3.2 (7)
C6—N4—C2—C1	178.3 (5)	N3—C13—C14—C15	2.2 (9)
Mn1—N4—C2—C1	−4.1 (6)	N1—C13—C14—C15	−175.9 (6)
N2—C1—C2—N4	176.9 (5)	N6—C14—C15—C16	0.2 (10)
N1—C1—C2—N4	−3.1 (7)	C13—C14—C15—C16	179.3 (6)
N2—C1—C2—C3	−4.8 (8)	C14—C15—C16—C17	−0.2 (11)
N1—C1—C2—C3	175.1 (5)	C15—C16—C17—C18	0.1 (12)
N4—C2—C3—C4	−0.4 (9)	C14—N6—C18—C17	0.2 (9)
C1—C2—C3—C4	−178.5 (5)	Mn1—N6—C18—C17	175.0 (5)
C2—C3—C4—C5	0.6 (9)	C16—C17—C18—N6	−0.2 (11)

### Hydrogen-bond geometry (Å, °)

**Table:**

*D*—H···*A*	*D*—H	H···*A*	*D*···*A*	*D*—H···*A*
C10—H10···Br1^i^	0.95	2.91	3.782 (6)	153.
C15—H15···Br1^ii^	0.95	2.91	3.744 (6)	148.

Symmetry codes: (i) −*x*, −*y*+2, −*z*; (ii) *x*−1, *y*, *z*.
